# MicroRNAs Mediated Regulation of Expression of Nucleoside Analog Pathway Genes in Acute Myeloid Leukemia

**DOI:** 10.3390/genes10040319

**Published:** 2019-04-24

**Authors:** Neha S. Bhise, Abdelrahman H. Elsayed, Xueyuan Cao, Stanley Pounds, Jatinder K. Lamba

**Affiliations:** 1 Department of Pharmacotherapy and Translational Research, Center for Pharmacogenomics, University of Florida, Gainesville, FL 32610, USA; Neha.bhise@gmail.com (N.S.B.); aelsayed@ufl.edu (A.H.E.); 2 Department of Experimental and Clinical Pharmacology, University of Minnesota, Minneapolis, MN 55455, USA; 3 Department of Acute and Tertiary Care, University of Tennessee Health Science Center, Memphis, TN 38163, USA; xcao12@uthsc.edu; 4 Department of Biostatistics, St. Jude Children’s Research Hospital, Memphis, TN 38105, USA; stanley.pounds@stjude.org

**Keywords:** nucleoside analogs, microRNAs, gene expression, drug resistance, AML

## Abstract

Nucleoside analog, cytarabine (ara-C) is the mainstay of acute myeloid leukemia (AML) chemotherapy. Cytarabine and other nucleoside analogs require activation to the triphosphate form (ara-CTP). Intracellular ara-CTP levels demonstrate significant inter-patient variation and have been related to therapeutic response in AML patients. Inter-patient variation in expression levels of drug transporters or enzymes involved in the activation or inactivation of cytarabine and other analogs is a prime mechanism contributing to development of drug resistance. Since microRNAs (miRNAs) are known to regulate gene-expression, the aim of this study was to identify miRNAs involved in regulation of messenger RNA expression levels of cytarabine pathway genes. We evaluated miRNA and gene-expression levels of cytarabine metabolic pathway genes in 8 AML cell lines and The Cancer Genome Atlas (TCGA) data base. Using correlation analysis and functional validation experiments, our data demonstrates that miR-34a-5p and miR-24-3p regulate DCK, an enzyme involved in activation of cytarabine and DCTD, an enzyme involved in metabolic inactivation of cytarabine expression, respectively. Further our results from gel shift assays confirmed binding of these mRNA-miRNA pairs. Our results show miRNA mediated regulation of gene expression levels of nucleoside metabolic pathway genes can impact interindividual variation in expression levels which in turn may influence treatment outcomes.

## 1. Introduction

Nucleoside analogs (NA) are a class of chemotherapeutic agents that structurally resemble the endogenous purine or pyrimidine nucleosides. These therapeutic agents mimic the endogenous nucleosides with respect to their uptake and metabolism and are incorporated into the newly synthesized DNA leading to inhibition of DNA synthesis and chain termination. Some of the nucleoside analogs also inhibit or block the enzymes that are required for the synthesis of purine or pyrimidine nucleotides and RNA synthesis, leading to the activation of the caspase cascade and cell death. The nucleoside analogs are extensively used for the treatment of both hematological malignancies and solid tumors. The pyrimidine nucleoside analog, cytarabine, is one of the most widely used chemotherapeutic drugs for the treatment of acute myeloid leukemia (AML). 

One of the major obstacles in the treatment of AML is development of resistance to nucleoside analogs. There is a growing need to understand the mechanisms that lead to development of resistance to these nucleoside analogs in order to help identify strategies that would effectively treat patients with relapsing or refractory diseases. One of the primary mechanisms of resistance to nucleoside analogs is insufficient intracellular concentration of the active triphosphate metabolite. This insufficient triphosphate levels could be due to inefficient cellular uptake of the drug, reduced levels of the activating enzyme, increased levels of inactivating enzymes and/or due to increased levels of endogenous deoxynucleotide (dNTP) pools [1,2,3,4]. Resistance could also develop due to inability to achieve sufficient alterations in the DNA strands or the dNTP pools, either due to altered interaction with DNA polymerases or by a lack of inhibition of ribonucleotide reductases, or due to inadequate p53 exonuclease activity. Since the expression and activity of drug transporters and metabolizing enzymes in the activation pathway of nucleoside analogs plays an important role in development of resistance to the NAs, it is essential to understand the factors influencing the expression and activity of these proteins.

MicroRNAs (miRNAs) are a group of novel gene regulators, which have been recently recognized to play an important role in cancers due to their tumor suppressive and oncogenic functions [5]. MiRNAs are known to regulate the expression of the target genes by binding to the specific sequence mainly on the 3’ untranslated region on the genes. Role of miRNAs in regulating the expression of various drug-metabolizing enzymes like cytochrome P450 3A4 (CYP3A4) etc., drug transporters like BCRP and various drug targets [6,7,8] have been established. However, there have not been any studies that have comprehensively evaluated the effect of miRNAs on the important genes involved in the transport, activation and inactivation of nucleoside analogs. Hence, the aim of this study was to assess the effect of miRNAs on the expression of nucleoside analog pharmacokinetic (PK) and pharmacodynamic (PD) pathway genes and in turn assessing their potential impact on resistance to nucleoside analogs. 

## 2. Materials and Methods

### 2.1. Cell culture and Reagents

The AML cell lines HL-60, MV-4-11, Kasumi-1, THP-1, AML-193 and KG-1 cell lines were obtained from American Type Culture Collection (ATCC) (Manassas, VA, USA), while MOLM-16 and ME-1 cell lines were obtained from DSMZ (Braunschweig, Germany). Kasumi-1, ME-1 and MOLM-16 cell lines were cultured in Roswell Park Memorial Institute (RMPI)-1640 medium supplemented with 20% fetal bovine serum (FBS), THP-1 cell line was cultured in RPMI-1640 medium supplemented with 10% FBS, HL-60 and KG-1 cell lines were cultured in IMDM medium supplemented with 20% FBS, while AML-193 cell lines was cultured in IMDM medium supplemented with 5% FBS, 0.005 mg/mL insulin, 0.005 mg/mL transferrin and 5 ng/mL granulocyte/macrophage colony stimulating factor (GM-CSF). All the cell lines were maintained in a 37 °C humidified incubator with 5% CO_2_. The cells were passaged every two to three days in order to maintain them in logarithmic growth phase.

### 2.2. RNA Isolation 

Total RNA was isolated from the AML cell pellets using RNeasy Plus Mini Kit (Qiagen, Valencia, CA, USA) according to the manufacturer’s protocol and stored in −80 °C until further analysis. The RNA quality and concentration were measured using NanoDrop 2000 UV-Vis spectrophotometer (Thermo Scientific, Wilmington, DE, USA). The ratio of absorbance at 260 nm and 280 nm was used to assess RNA sample purity and A260/A280 ratio of 1.8–2.1 was considered to be indicative of highly purified RNA. RNA was normalized to 0.2 μg/μL with nuclease-free water before being used for performing reverse transcription reactions, as recommended by the manufacturer. The total RNA was reverse transcribed to complementary DNA (cDNA) using High Capacity cDNA Reverse Transcription Kit (Applied Biosystems, Foster City, CA, USA) according to manufacturer’s protocol.

### 2.3. Gene Expression Analysis

The expression of nucleoside analog genes was determined using the TaqMan^®^ Low Density Array (TLDA) cards (Applied Biosystems). Each TLDA card was custom designed with pre-loaded gene expression assays for measuring the messenger RNA (mRNA) expression of selected nucleoside analog metabolic pathway genes- (*n* = 14) deoxycytidine kinase (*DCK*), cytidine deaminase (*CDA*), solute carrier family 29, member 1 (*SLC29A1*), solute carrier family 28, member 1 (SLC28A1), solute carrier family 28, member 3 (SLC28A3), deoxycytidylate deaminase (*DCTD*), 5’-nucleotidase, cytosolic II (*NT5C2*), 5’-nucleotidase cytosolic III (*NT5C3*), cytidine 5’-triphosphate synthase (CTPS), cytidine monophosphate kinase (*CMPK*), nucleoside diphosphate kinase 1 (*NME1*), ribonucleotide reductase M1 (*RRM1*), ribonucleotide reductase M2 (*RRM2*), ribonucleotide reductase M2B (*RRM2B*). Each TLDA card consists of eight separate loading ports that fill into 48 separate wells, for a total of 384 wells per card. Thus, each card could analyze the expression of 24 different genes for eight different samples in duplicates. Each cDNA sample was added to equal volume of 2X TaqMan Universal PCR Master Mix (Thermo Scientific) and 100 μL of the sample-specific PCR mix was added to the fill reservoir on the TLDA card. The card was centrifuged twice for one minute at 1200 rpm to distribute the sample-specific PCR reaction mix to the reaction wells. The card was sealed using the TaqMan Array Micro Fluidic Card Sealer (Thermo Scientific) and placed on microfluidic card thermal cycling block of Applied Biosystems 7900HT Fast Real-time PCR System (Applied Biosystems). Thermal cycling conditions were as follows: 2 min at 50 °C, 10 min at 94.5 °C, 30 s at 97 °C, 1 min at 59.7 °C for 40 cycles. The target mRNA expression levels were normalized to GAPDH and the expression values of nucleoside analogs pathway genes were calculated using ∆∆C_T_ method [9].

### 2.4. MicroRNA Expression Analysis 

For determination of miRNA expression, total RNA was isolated using mirVana™ miRNA Isolation kit (Life Technologies, Carlsbad, CA, USA) as per the manufacturer’s protocol. The RNA quality and concentration were measured using NanoDrop 2000 UV-Vis spectrophotometer (Thermo Scientific). A total of 100 ng of purified total RNA was used for nCounter miRNA sample preparation reactions according to manufacturer’s instructions and was assayed for determination of 800 human miRNA expression using the nCounter Human v2 miRNA Expression Assay kit (Nanostring Technologies, Seattle, WA, USA). Preparation of small RNA samples involved multiplexed ligation of specific tags (miRtags) to the target miRNAs that provide unique identification for each miRNA species. After ligation, the detection was done by hybridization to microRNA: tag specific nCounter capture and barcoded reporter probes. Data collection was carried out using the nCounter Digital Analyzer (Nanostring Technologies) at The University of Minnesota Genomics Center, following manufacturer’s instructions to count individual fluorescent barcodes and quantify the target miRNA molecules present in each sample. MiRNA expression data normalization was performed using the nSolver™ Analysis Software (Nanostring Technologies) according to the manufacturer’s instructions. In particular, initially the data was normalized using the expression of the top 100 code sets. Further, to account for the background correction, mean of negative controls plus two-standard deviation (SD) method was used. In order to avoid using the miRNAs with a very low expression, we further filtered out the miRNAs with expression counts < 30 (2 times the mean ± 2 SD of negative control value), in order to account for the background noise. Total 412 miRNAs with expression counts > 30 were included for further analysis.

### 2.5. Acute Myeloid Leukemia Patient Sample Data from The Cancer Genome Atlas

The miRNA expression and mRNA expression of the nucleoside analog pathway genes in AML patients was extracted from The Cancer Genome Atlas (TCGA) Data Portal (cancergenome.nih.gov) [10]. Out of the 200 AML patients in TCGA database, 197 patients had gene expression profiling data available and 187 patients had miRNA expression data available. 186 patients had both gene expression and miRNA expression data available.

### 2.6. Electrophoretic Mobility Shift Assays

The functional validation for binding efficiencies between miRNAs and mRNAs was performed using the electrophoretic mobility shift assays (EMSAs). The binding free energy between the respective mRNA and miRNA pair was predicted using the RNAhybrid software. The miRNA oligonucleotides were labeled with cy5™ dye on their 5’ ends. The 2’ O-methyl-modified mRNA oligonucleotides were labeled with IRDye^®^800 (LI-COR Biosciences, Lincoln, NE, USA) dye on their 5’ ends. The labeled oligonucleotides were synthesized by Integrated DNA Technologies (Coralville, IA, USA). RNA EMSA experiment was performed using the LightShift Chemiluminescent RNA EMSA Kit (Thermo Scientific) according to the manufacturer’s protocol. The mRNA oligonucleotide was heated for 10 min at 80 °C and then placed on ice in order to relax the secondary structures. In each 20 μL binding reaction, 200 nM miRNA oligonucleotide and/or mRNA oligonucleotide were mixed with RNA EMSA binding buffer and incubated at 25 °C for 25 min. The reaction mixtures were separated on a 12% polyacrylamide gel by electrophoresis at 4 °C. The binding reactions were transferred onto nylon membrane and the resulting mobility shifts were imaged using and Odyssey CLx Infrared System (LI-COR Biosciences).

### 2.7. Bioinformatic Analysis

Prediction of miRNA binding sites was performed using multiple prediction programs, which use different criteria for prediction of binding sites: TargetScan (www.targetscan.org), miRanda (www.microRNA.org), PICTAR (pictar.mdc-berlin.de), miRWalk (www.umm.uni-heidelberg.de/apps/zmf/mirwalk). Binding free energy calculations were performed using RNAhybrid software [11]. The 3’UTR (3’ untranslated region) sequence of mRNA was obtained from the UCSC Genome browser (https://genome.ucsc.edu/) and miRNA sequence was obtained from miRBase software (http://www.mirbase.org/).

### 2.8. Statistical Analysis 

The nonparametric Spearman correlation was used to measure the correlation of mRNA expression with miRNA expression. Statistical significance was determined when *p*-value was < 0.01.

## 3. Results

### 3.1. Effect of Micro RNA on the Expression of Nucleoside Analog Pathway Genes in Acute Myeloid Leukemia Cell Lines 

**Figure 1:**
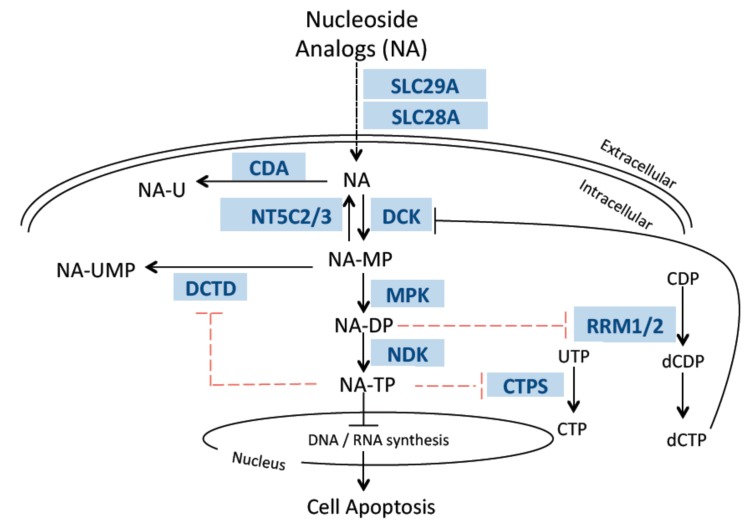
Disposition pathway of nucleoside analogs, cytarabine and clofarabine. DCTD: Deoxycytidylate deaminase, DCK: Deoxycytidine kinase, CDA: Cytidine deaminase, NT5C2/3: 5’-Nucleotidase, cytoplasmic, CTPS: CTP synthase, RRM1/2: Ribonucleotide reductase, SLC29A: Solute carrier family 29, SLC28A: Solute carrier family 28, MPK: Monophosphate kinase, NDK: Nucleoside diphosphate kinase

**Figure 2:**
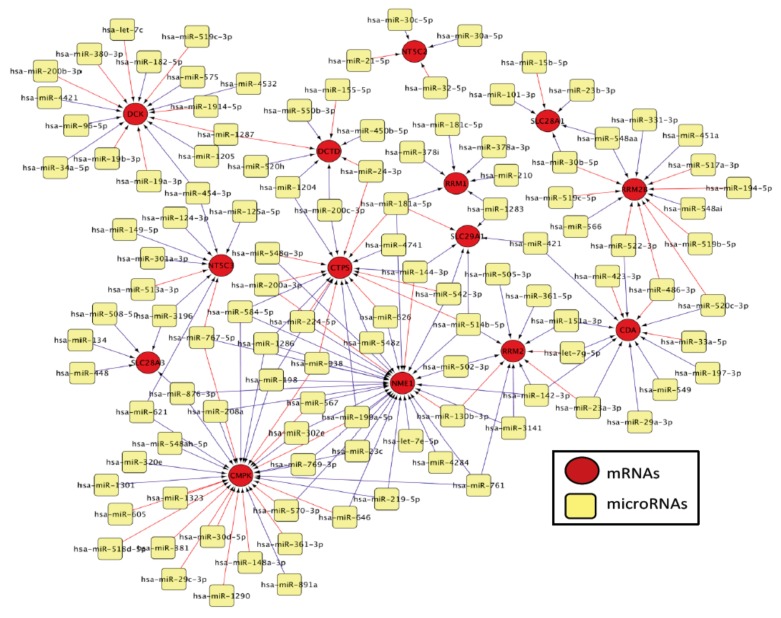
MicroRNA-mRNA network constructed using CyTargetLinker [12]. MiRNAs associated with the nucleoside analog metabolic pathway genes are shown in yellow and the mRNAs are depicted in red. Blue lines indicated positive and red indicated negative associations.

We determined expression of 800 miRNAs and 13 genes involved in PK/PD pathway of nucleoside analogs (Figure 1) in cytogenetically different AML cell lines (*n* = 8). In order to identify the miRNAs associated with the expression of nucleoside analog pathway genes, we correlated the miRNA expression and mRNA expression using the spearman correlation analysis. Table 1 lists the negative correlations of nucleoside analog pathway genes and respective miRNAs at *p* <0.01. We further used CyTargetLinker software [12] to establish the network of miRNAs correlated with the respective PK/PD pathway genes of the nucleoside analogs. Figure 2 shows the miRNA-mRNA pairs identified by CyTargetLinker.

**Figure 3:**
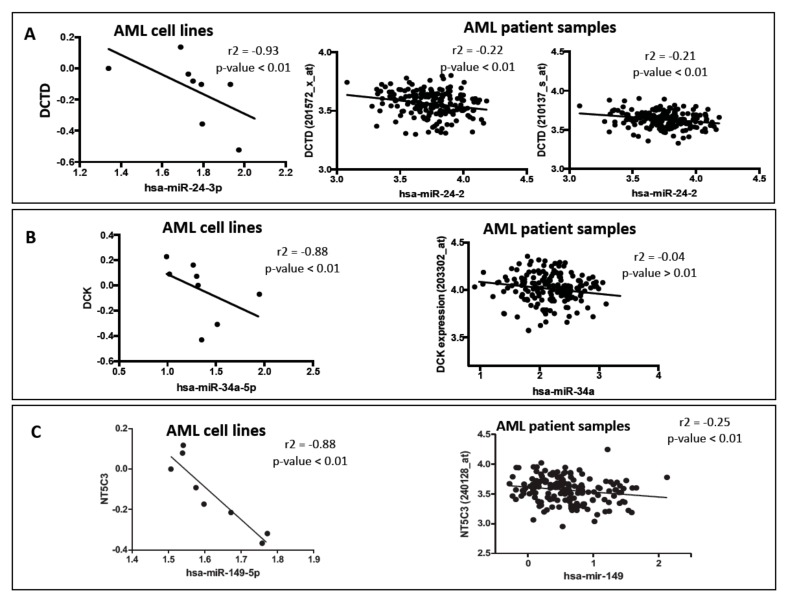
Correlation of miRNAs with nucleoside analog pathway gene expression in acute myeloid leukemia (AML) cell lines and patient samples from The Cancer Genome Atlas (TCGA) database. (**A**) Correlation between DCTD and hsa-miR-24a-3p. (**B**) Correlation between DCK and hsa-miR-34a-5p. (**C**) Correlation between NT5C3 and hsa-miR-149.

The expression of *DCK* (the rate-limiting enzyme in the nucleoside analog pathway) correlated with the expression of miR-34a-5p expression (spearman *r* = −0.88; *p*-value < 0.01) and miR-96-5p expression (spearman *r* = −0.91; *p*-value < 0.01). The expression of deactivating enzyme *DCTD* was found to be correlated with miR-24-3p expression (spearman *r* = −0.93; p-value < 0.01). Interestingly, in our previous study, we identified that expression of miRNA miR-24-3p was correlated with cytarabine-induced cell cytotoxicity (spearman *r* = −0.81, *p*-value < 0.05) [13] which is in agreement with the current observation. The expression of *CMPK*, a kinase responsible for phosphorylation of the monophosphate form of nucleoside analog was negatively correlated with the expression of miR-1301, miR-1323, miR-320e, miR-381, miR-507, miR-584-5p, miR-605, miR-762, miR-769-3p, miR-891a (all *p*-values < 0.01). RRM2 expression was found to be negatively associated with the expression of miR-151a-3p (*p*-value < 0.01). Figure 3 shows correlation plots between *DCTD*-miR-24, *DCK*-miR34 and *NT5C3*-miR149 pairs.

### 3.2. Bioinformatic Prediction of Binding of Micro RNAs and Messenger RNAs

MiRNAs are known to guide the RNA-induced silencing complex (RISC) to the specific sequence (usually in located in the 3’UTR) on the target mRNA. Using various bioinformatic prediction programs (TargetScan, miRanda, PICTAR, miRWalk) we determined if the miRNAs that were correlated with gene expression had binding sites on 3’-UTR of target genes. MiRNAs miR-1323, miR-30d-5p, miR-381, and miR-605 were predicted to have binding sites on *CMPK* gene, while miRNA miR-24-3p was found to have binding site on *DCTD* by multiple prediction programs. Supplementary Figure S1 shows comparisons of different miRNA prediction programs for genes involved in nucleoside analog pathways.

### 3.3. Effect of Micro RNAs on the Expression of Nucleoside Analog Pathway Genes in Acute Myeloid Leukemia Patients

In order to validate the significant correlations between miRNAs and mRNAs identified in AML cell lines, we evaluated the correlation between miRNA expression and nucleoside analog pathway gene expression in AML patient samples from TCGA database (*n* = 186). We extracted the miRNA expression data and nucleoside analog pathway gene expression data for AML patients from TCGA database and performed spearman correlation to identify the significant mRNA-miRNA pairs. Consistent with results from AML cell lines miR-24-3p was inversely correlated with the expression of both the probes for *DCTD* in AML patient samples (*r* = −0.22; *p*-value < 0.01 and *r* = −0.21; *p*-value < 0.01, Figure 3A). *DCK*-mir-34a pair unfortunately did not show significant association within the TCGA data-set. miR-149 was significantly correlated with expression of *NT5C3* in AML patients (*r* = −0.25; *p*-value < 0.01, Figure 3C).

#### Validation of Binding Efficiencies between Messenger RNAs and Micro RNAs 

**Figure 4:**
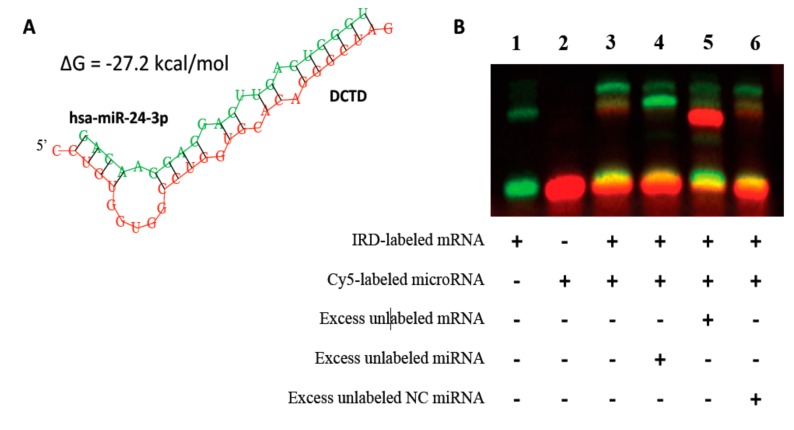
Validation of binding interaction between DCTD mRNA and has-miR-24-3p by RNA electrophoretic mobility shift assays (EMSAs). RNA EMSA with cy5-labeled has-miR-24-3p oligonucleotide and 2’-O-methyl modified and IRD-800 labeled DCTC mRNA oligonucleotide. Lanes 1 and 2 show the mobility of the labeled mRNA or miRNA oligonucleotide. Lane 3 shows the mobility of the labeled has-miR-24-3p oligonucleotide with *DCTD* mRNA oligonucleotide. Lanes 4 and 6 show the mobility of labeled *DCTD* mRNA oligonucleotide in presence of unlabeled excess specific competitor (has-miR-24-3p) ad excess unlabeled non-specific competitor (NC).

**Figure 5:**
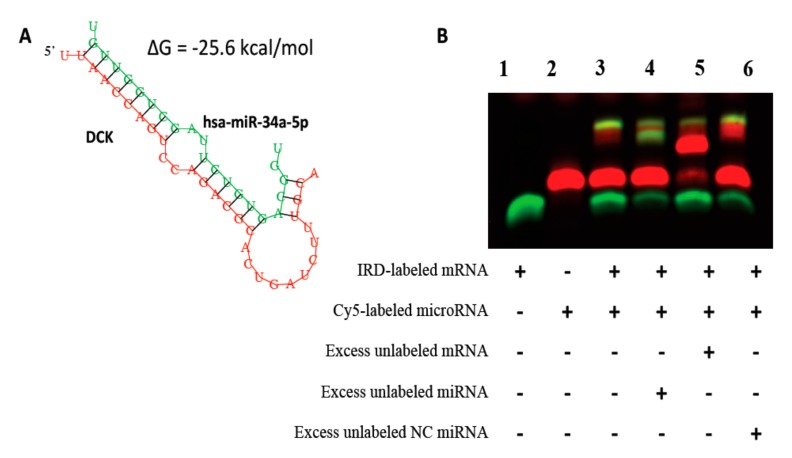
Validation of binding interactions between *DCK* and mRNA and has-miR-34a-5p by RNA EMSAs. RNA EMSA with cys5-labeled has-miR-34a-5p oligonucleotide and 2’-O-methyl modified and IRD-800 labeled DCK mRNA oligonucleotide. Lanes 1 and 2 show the mobility of the labeled mRNA or miRNA oligonucleotide. Lane 3 shows the mobility of the labeled has-miR-34a-5p oligonucleotide with DCK mRNA oligonucleotide. Lanes 4 and 6 show the mobility of labeled DCK mRNA oligonucleotide in presence of unlabeled excess specific competitor (has-miR-34a-5p) and excess unlabeled non-specific competitor (NC).

MiRNAs are known to bind to the specific seed sequence on the 3’UTR of the mRNAs, thereby regulating the expression of their target genes. In order to validate the binding between the mRNA-miRNAs identified from the in vitro studies and in AML patient samples, we performed RNA EMSA assays. We validated the interaction between *DCTD* and miR-24, since we identified this mRNA-miRNA pair to be significantly inversely correlated in both AML cell line and AML patient samples. In addition, various prediction databases predicted miRNA miR-24 to have binding site on the 3’UTR of *DCTD* mRNA. In silico analysis predicted miR-24-3p and miR-34a-5p might form complexes with target sequences in the 3’UTR of *DCTD* and *DCK* respectively with minimum free energies of binding of −27.2 kcal/mol for *DCTD* and miR-24-3p (Figure 4A and Table 1) and -25.6 kcal/mol for *DCK* and miR-34a-5p (Figure 5A and Table 1). The RNA EMSA results for IRD-800^®^-labeled *DCTD* and Cy-5-labeled miR-24-3p show miR-24a-3p was able to bind to its target sequence on *DCTD* 3’UTR (Figure 4B, lane 3) as seen by the band shift. The thermodynamic stability of this complex correlated with binding observed in the RNA EMSA assays. In addition, we found that mRNA-miRNA complex formed by *DCTD* and miR-24a-3p could be eliminated by adding excess unlabeled hsa-miR-24a-3p probe (Figure 4B, lane 4), but not by adding excess unlabeled non-specific probe (Figure 4B, lane 6). Adding excess unlabeled mRNA probe resulted in binding of all the labeled miRNA giving a greater intensity signal (Figure 4B, lane 5).

Similar to the interaction observed between *DCTD* and miR-24a-3p, we observed that miR-34a-5p binds to *DCK* 3’UTR as seen by shift in the band (Figure 5B, lane 3) and this interaction was eliminated by addition of unlabeled probe (Figure 5B, lane 4). Since the minimum free energy of binding for *NT5C3*- miR-149 pair was not strong (Table 1), we did not pursue EMSA assays for this pair. 

We further evaluated the association of the in vitro chemosensitivity of ara-C (defined previously Bhise et al, 2015 [13] with the three top miRNAs in the AML cell lines. As shown in Supplementary Figure S2. consistent with our results of miR-24-*DCTD* pair, we observed cell lines that were sensitive to ara-C has significantly higher levels of miR-24 as compared to cell lines that are resistant to ara-C (*p* = 0.03). These results suggest that high miR-24 in ara-C sensitive cell lines might be resulting in lowering *DCTD* levels and given that *DCTD* is involved in inactivation of ara-C, its low levels will result in better response.

## 4. Discussion

Nucleoside analogs are synthetic analogs of endogenous nucleosides that largely used for the treatment of hematological malignancies and solid tumors. Cytarabine, a pyrimidine nucleoside analog is the backbone of AML chemotherapy, while clofarabine is a second-generation purine nucleoside analog that is currently being investigated for treatment of AML in various clinical trials. Both cytarabine and clofarabine require active transport into the cell by nucleoside transporters, followed by activation by various kinases to form active di- and tri-phosphate metabolites that are incorporated in growing DNA strand and/or inhibit various enzymes involved in synthesis of endogenous nucleotides (Figure 1). However, despite being the backbone of treatment regimen used in AML patients, there is variability in response to cytarabine and other nucleoside analogs. In our previous study, we have demonstrated that miRNA expression is predictive of response to cytarabine therapy in AML patients and is also significantly associated with in vitro chemosensitivity of cytarabine in AML cell lines [13]. Recent studies are expanding on therapeutic relevance of miRNAs in AML. Recent review article by Wallace et al, 2017, have summarized the miRNAs that are deregulated in AML and thus hold potential as AML biomarkers. Further, mimics for miR-22, miR29b and miR-181 and antagomiRs for miR-21/miR-196b and miR-126 are currently under investigation for their therapeutic potential in AML [14]. However, given that cytarabine a nucleoside analog is the mainstay of AML chemotherapy for decades, in the current study, we wanted to determine if miRNA mediated regulation of expression of the transporters, activating and inactivating genes involved in the metabolic pathway of nucleoside analogs, could contribute to development of resistance to nucleoside analogs in AML patients. We hypothesized that miRNAs could bind to the 3’UTR of the mRNAs of nucleoside analog metabolic pathway genes, thereby altering their expression, which in turn would result in lower intracellular levels of active nucleoside analog triphosphate, resulting in chemo-resistance.

In our current study, DCK expression was negatively correlated with the expression of hsa-miR-34a-5p and hsa-miR-96-5p in AML cell lines (*p*-value < 0.01). DCK is a rate-limiting enzyme that is involved in activation of cytarabine and clofarabine. Studies have reported that decreased or complete loss of DCK activity results in cellular resistance to cytarabine [15,16,17]. Also, *DCK* mRNA expression has been shown to be positively associated with AML patient outcome, AML patients with higher *DCK* mRNA expression demonstrated longer event-free survival than those with lower *DCK* mRNA expression [18]. Using EMSA, we were able to show that hsa-miR-34a-5p by binding to the 3’-UTR regulates expression of *DCK*. MiRNA hsa-miR-34a has been extensively studied in various cancers [19,20,21,22,23] and it has been shown to play an important role as a tumor suppressor by targeting various genes. However, the effect of miR-34a on *DCK* expression has not yet been studied. Identification of this additional regulatory mechanism for an important enzyme in the activation of nucleoside analogs could help in better prediction of chemosensitivity of these drugs. Unfortunately, we did not observe any significant correlation between *DCK* mRNA and miR-34a levels in TCGA dataset. 

We also identified hsa-miR-24-3p to be negatively correlated with the expression of *DCTD* in both AML cell lines and in AML patient samples (*p*-value < 0.01). In addition, multiple bioinformatic prediction programs identified a binding site for hsa-miR-24-3p on the 3’UTR of DCTD. Our RNA EMSA results confirmed the binding interaction between DCTD and miR-24-3p. DCTD is an enzyme involved in deamination of the monophosphate form of the nucleoside analog, thus inactivating the drug. The levels of DCTD could thus affect the levels of the intracellular active triphosphate metabolites of nucleoside analogs. However, the role of *DCTD* in chemosensitivity of nucleoside analogs is poorly defined. Various studies have demonstrated a significant role of *DCTD* in metabolism of the monophosphate metabolite of the nucleoside analogs in human leukemia cells [24,25,26]. Sequencing of this gene identified a nonsynonymous SNP affecting the activity of *DCTD* in vitro [27]. Hence, the limited data on the regulation of *DCTD* gene warrants the need to evaluate additional mechanism regulating gene-expression. MiRNA miR-24 is has also been extensively studied in various cancers [28,29,30,31,32,33,34] and has been shown to enhance metastasis and invasion. Increased expression of miR-24 has been associated with increased risk of relapse and poor survival in acute lymphoblastic leukemia (ALL) [32]. In addition to miR-24 and hsa-miR-34a-5p, we also identified multiple other miRNAs that correlated with multiple genes in the PK/PD pathway (Table 1). We acknowledge the limitation of use of different platforms of miRNA and mRNA quantification between cell lines and the TCGA data-set which warrant the need for future prospective study to validate our results. Additionally, given that gel-shift assays do not consider interactions with argonaute which can impact thermodynamics of target recognition future in-depth mechanistic validation considering these factors are needed to establish miRNA-mRNA regulatory pairs of significant therapeutic implications in AML.

In summary, we identified several miRNAs, which were significantly associated with the expression of nucleoside analog pathway genes. Identification of these additional mechanisms of regulation would help provide a better understanding of the variability in the expression of these enzymes and transporters and in turn, help in better prediction of therapeutic response in AML patients. While additional functional studies are required to gain mechanistic understanding of these miRNA-mRNA interactions and its effect on the protein levels and activity, this study helps identify candidate miRNAs for further studies.

## Figures and Tables

**Table 1:** MiRNAs with significant negative association (as reflected by *r* value) with nucleoside analog pathway genes in AML Cell lines.

Pathway Genes	MiRNAs	Spearman *r*	*p* Value	Minimum Free Energy (mfe) mRNA-miRNA Pair (kcal/mol)
*DCTD*	hsa-miR-24-3p	−0.9341	0.0011	−27.2
*DCK*	hsa-miR-96-5p	−0.9048	0.0046	−25.6
*DCK*	hsa-miR-34a-5p	−0.881	0.0072	−24.3
*NT5C3*	hsa-miR-149-5p	−0.881	0.0072	−21.4
*RRM2*	hsa-miR-151a-3p	−0.9524	0.0011	−26.4
*RRM2B*	hsa-miR-194-5p	−0.881	0.0072	−21.6
*CMPK*	hsa-miR-1301	−0.9762	0.0004	−23.6
*CMPK*	hsa-miR-320e	−0.9524	0.0011	−20.1
*CMPK*	hsa-miR-1323	−0.9286	0.0022	−22.3
*CMPK*	hsa-miR-584-5p	−0.9286	0.0022	−23.7
*CMPK*	hsa-miR-381	−0.881	0.0072	−25.8
*CMPK*	hsa-miR-507	−0.881	0.0072	−19.1
*CMPK*	hsa-miR-605	−0.881	0.0072	−23.1
*CMPK*	hsa-miR-762	−0.881	0.0072	−29.2
*CMPK*	hsa-miR-769-3p	−0.881	0.0072	−27.6
*CMPK*	hsa-miR-891a	−0.881	0.0072	−22.9
*NME1*	hsa-miR-514b-5p	−0.9286	0.0022	−22
*NME1*	hsa-miR-542-3p	−0.9286	0.0022	−19.3
*NME1*	hsa-miR-570-3p	−0.9048	0.0046	−22.2
*NME1*	hsa-miR-646	−0.9048	0.0046	−25.9
*NME1*	hsa-miR-224-5p	−0.881	0.0072	−17.9
*NME1*	hsa-miR-761	−0.881	0.0072	−24.3
*NME1*	hsa-miR-767-5p	−0.881	0.0072	−28.8
*SLC28A1*	hsa-miR-548aa	−0.9643	0.0028	−16.3
*SLC28A3*	hsa-miR-448	−0.9643	0.0028	−23.8

## Supplementary Materials

The following are available online at https://www.mdpi.com/2073-4425/10/4/319/s1, Figure S1: Comparison of microRNA prediction programs for predicting binding sites on nucleoside analog pathway genes. Figure S2: A) Eight AML cell lines were treated with varying concentration of ara-C for 48 hrs followed by measuring cell viability using MTT assays using as described previously (Bhise et al, 2015). Area under the survival was calculated using Graphpad Prism and cell lines were classified as sensitive or resistant to ara-C. B) miR24-3p levels were observed to be higher in cell lines sensitive to ara-C as compared to ara-C resistant cell lines.

## References

[1] Galmarini C.M., Clarke M.L., Jordheim L., Santos C.L., Cros E., Mackey J.R., Dumontet C. (2004). Resistance to gemcitabine in a human follicular lymphoma cell line is due to partial deletion of the deoxycytidine kinase gene. BMC Pharmacol..

[2] Galmarini C.M., Thomas X., Calvo F., Rousselot P., Rabilloud M., El Jaffari A., Cros E., Dumontet C. (2002). In vivo mechanisms of resistance to cytarabine in acute myeloid leukaemia. Br. J. Haematol..

[3] Lotfi K., Juliusson G., Albertioni F. (2003). Pharmacological basis for cladribine resistance. Leuk. Lymphoma.

[4] Mansson E., Flordal E., Liliemark J., Spasokoukotskaja T., Elford H., Lagercrantz S., Eriksson S., Albertioni F. (2003). Down-regulation of deoxycytidine kinase in human leukemic cell lines resistant to cladribine and clofarabine and increased ribonucleotide reductase activity contributes to fludarabine resistance. Biochem. Pharmacol..

[5] Kent O.A., Mendell J.T. (2006). A small piece in the cancer puzzle: MicroRNAs as tumor suppressors and oncogenes. Oncogene.

[6] Rieger J.K., Reutter S., Hofmann U., Schwab M., Zanger U.M. (2015). Inflammation-associated microRNA-130b down-regulates cytochrome P450 activities and directly targets CYP2C9. Drug Metab. Dispos..

[7] Pan Y.Z., Morris M.E., Yu A.M. (2009). MicroRNA-328 negatively regulates the expression of breast cancer resistance protein (BCRP/ABCG2) in human cancer cells. Mol. Pharmacol..

[8] Mishra P.J., Humeniuk R., Mishra P.J., Longo-Sorbello G.S., Banerjee D., Bertino J.R. (2007). A miR-24 microRNA binding-site polymorphism in dihydrofolate reductase gene leads to methotrexate resistance. Proc. Natl. Acad. Sci. USA.

[9] Livak K.J., Schmittgen T.D. (2001). Analysis of relative gene expression data using real-time quantitative PCR and the 2^−ΔΔC^_T_ Method. Methods.

[10] Cancer Genome Atlas Research Network (2013). Genomic and epigenomic landscapes of adult de novo acute myeloid leukemia. N. Engl. J. Med..

[11] Kruger J., Rehmsmeier M. (2006). RNAhybrid: microRNA target prediction easy, fast and flexible. Nucleic Acids Res..

[12] Kutmon M., Kelder T., Mandaviya P., Evelo C.T., Coort S.L. (2013). CyTargetLinker: A cytoscape app to integrate regulatory interactions in network analysis. PLoS ONE.

[13] Bhise N.S., Chauhan L., Shin M., Cao X., Pounds S., Lamba V., Lamba J.K. (2015). MicroRNA-mRNA Pairs Associated with outcome in AML: From in vitro cell-based studies to AML patients. Front. Pharmacol..

[14] Wallace J.A., O’Connell R.M. (2017). MicroRNAs and acute myeloid leukemia: Therapeutic implications and emerging concepts. Blood.

[15] Dumontet C., Fabianowska-Majewska K., Mantincic D., Callet Bauchu E., Tigaud I., Gandhi V., Lepoivre M., Peters G.J., Rolland M.O., Wyczechowska D. (1999). Common resistance mechanisms to deoxynucleoside analogues in variants of the human erythroleukaemic line K562. Br. J. Haematol..

[16] Bhalla K., Nayak R., Grant S. (1984). Isolation and characterization of a deoxycytidine kinase-deficient human promyelocytic leukemic cell line highly resistant to 1-β-d-arabinofuranosylcytosine. Cancer Res..

[17] Verhoef V., Sarup J., Fridland A. (1981). Identification of the mechanism of activation of 9-β-D-arabinofuranosyladenine in human lymphoid cells using mutants deficient in nucleoside kinases. Cancer Res..

[18] Galmarini C.M., Thomas X., Calvo F., Rousselot P., El Jafaari A., Cros E., Dumontet C. (2002). Potential mechanisms of resistance to cytarabine in AML patients. Leuk. Res..

[19] Garofalo M., Jeon Y.J., Nuovo G.J., Middleton J., Secchiero P., Joshi P., Alder H., Nazaryan N., di Leva G. (2015). Correction: MiR-34a/c-dependent PDGFR-α/β downregulation inhibits tumorigenesis and enhances TRAIL-induced apoptosis in lung cancer. PLoS ONE.

[20] Hong J.H., Roh K.S., Suh S.S., Lee S., Sung S.W., Park J.K., Byun J.H., Kang J.H. (2015). The expression of microRNA-34a is inversely correlated with c-MET and CDK6 and has a prognostic significance in lung adenocarcinoma patients. Tumour Biol..

[21] Lu G., Sun Y., An S., Xin S., Ren X., Zhang D., Wu P., Liao W., Ding Y., Liang L. (2015). MicroRNA-34a targets FMNL2 and E2F5 and suppresses the progression of colorectal cancer. Exp. Mol. Pathol..

[22] Qiao P., Li G., Bi W., Yang L., Yao L., Wu D. (2015). microRNA-34a inhibits epithelial mesenchymal transition in human cholangiocarcinoma by targeting Smad4 through transforming growth factor-beta/Smad pathway. BMC Cancer.

[23] Wang X., Li J., Dong K., Lin F., Long M., Ouyang Y., Wei J., Chen X., Weng Y., He T. (2015). Tumor suppressor miR-34a targets PD-L1 and functions as a potential immunotherapeutic target in acute myeloid leukemia. Cell. Signal..

[24] Capizzi R.L., White J.C., Powell B.L., Perrino F. (1991). Effect of dose on the pharmacokinetic and pharmacodynamic effects of cytarabine. Semin. Hematol..

[25] Fridland A., Verhoef V. (1987). Mechanism for ara-CTP catabolism in human leukemic cells and effect of deaminase inhibitors on this process. Semin. Oncol..

[26] Liliemark J.O., Plunkett W. (1986). Regulation of 1-β-D-arabinofuranosylcytosine 5′-triphosphate accumulation in human leukemia cells by deoxycytidine 5′-triphosphate. Cancer Res..

[27] Gilbert J.A., Salavaggione O.E., Ji Y., Pelleymounter L.L., Eckloff B.W., Wieben E.D., Ames M.M., Weinshilboum R.M. (2006). Gemcitabine pharmacogenomics: Cytidine deaminase and deoxycytidylate deaminase gene resequencing and functional genomics. Clin. Cancer Res..

[28] Xu L., Chen Z., Xue F., Chen W., Ma R., Cheng S., Cui P. (2015). MicroRNA-24 inhibits growth, induces apoptosis, and reverses radioresistance in laryngeal squamous cell carcinoma by targeting X-linked inhibitor of apoptosis protein. Cancer Cell Int..

[29] Lu K., Wang J., Song Y., Zhao S., Liu H., Tang D., Pan B., Zhao H., Zhang Q. (2015). miRNA-24-3p promotes cell proliferation and inhibits apoptosis in human breast cancer by targeting p27Kip1. Oncol. Rep..

[30] Manvati S., Mangalhara K.C., Kalaiarasan P., Srivastava N., Bamezai R.N. (2015). miR-24-2 regulates genes in survival pathway and demonstrates potential in reducing cellular viability in combination with docetaxel. Gene.

[31] Zhao G., Liu L., Zhao T., Jin S., Jiang S., Cao S., Han J., Xin Y., Dong Q., Liu X. (2015). Upregulation of miR-24 promotes cell proliferation by targeting NAIF1 in non-small cell lung cancer. Tumour Biol..

[32] Organista-Nava J., Gomez-Gomez Y., Illades-Aguiar B., del Carmen Alarcon-Romero L., Saavedra-Herrera M.V., Rivera-Ramirez A.B., Garzón-Barrientos V.H., Leyva-Vázquez M.A. (2015). High miR-24 expression is associated with risk of relapse and poor survival in acute leukemia. Oncol. Rep..

[33] Gao Y., Liu Y., Du L., Li J., Qu A., Zhang X., Wang L., Wang C. (2015). Down-regulation of miR-24-3p in colorectal cancer is associated with malignant behavior. Med. Oncol..

[34] Pan B., Chen Y., Song H., Xu Y., Wang R., Chen L. (2015). Mir-24-3p downregulation contributes to VP16-DDP resistance in small-cell lung cancer by targeting *ATG4A*. Oncotarget.

