# Supplementary material for: MicroRNAs Mediated Regulation of Expression of Nucleoside Analog Pathway Genes in Acute Myeloid Leukemia
Source: Genes (Basel). 2019 Apr 24;10(4):319. doi: 10.3390/genes10040319 (PMC6523677; doi:10.3390/genes10040319)
Supplement: Supplementary file 1 [file genes-10-00319-s001.pdf]

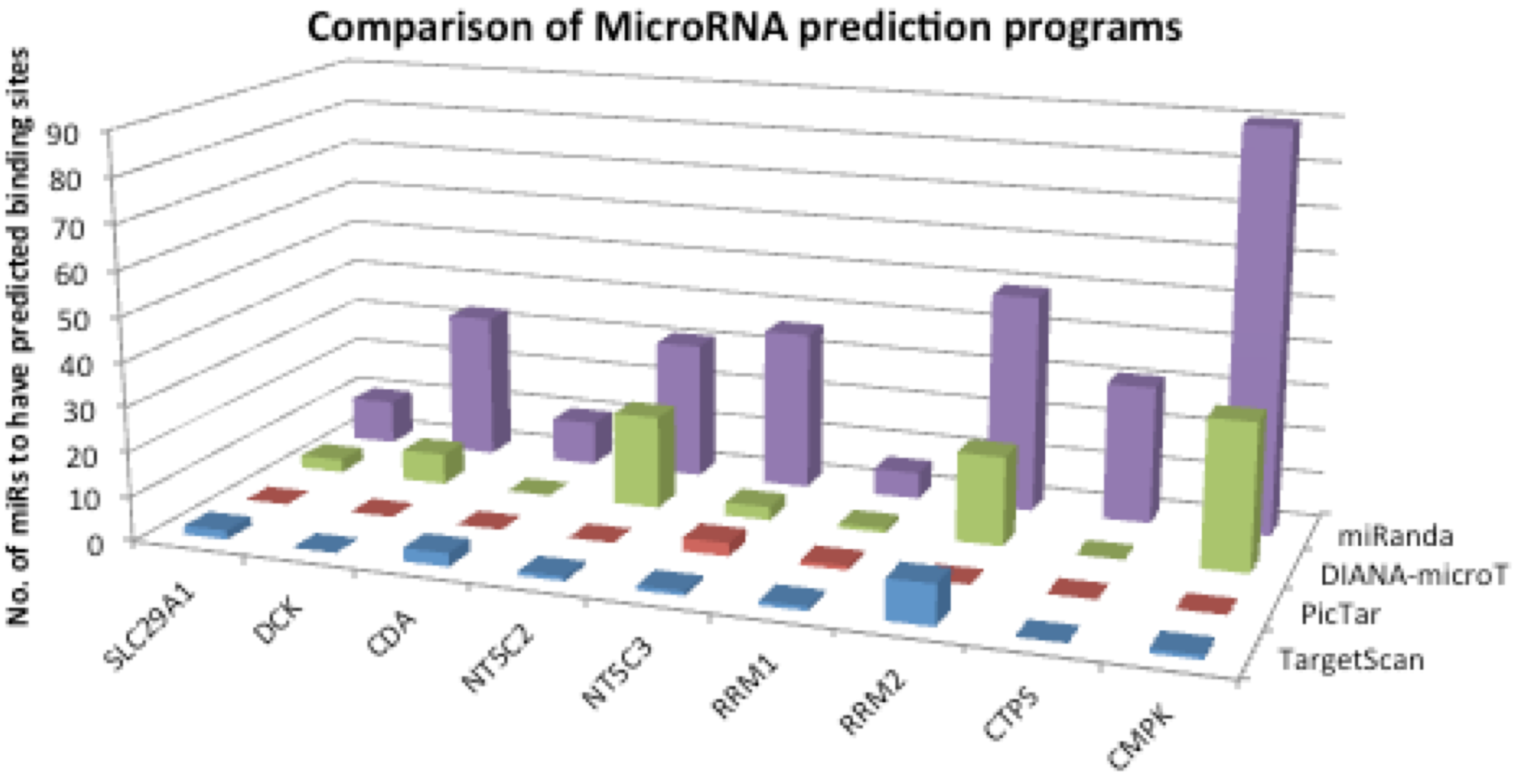

Supplementary figure 1: Comparison of microRNA prediction programs for predicting binding sites on nucleoside analog pathway genes

A) In vitro ara-C sensitivity of AML cell lines

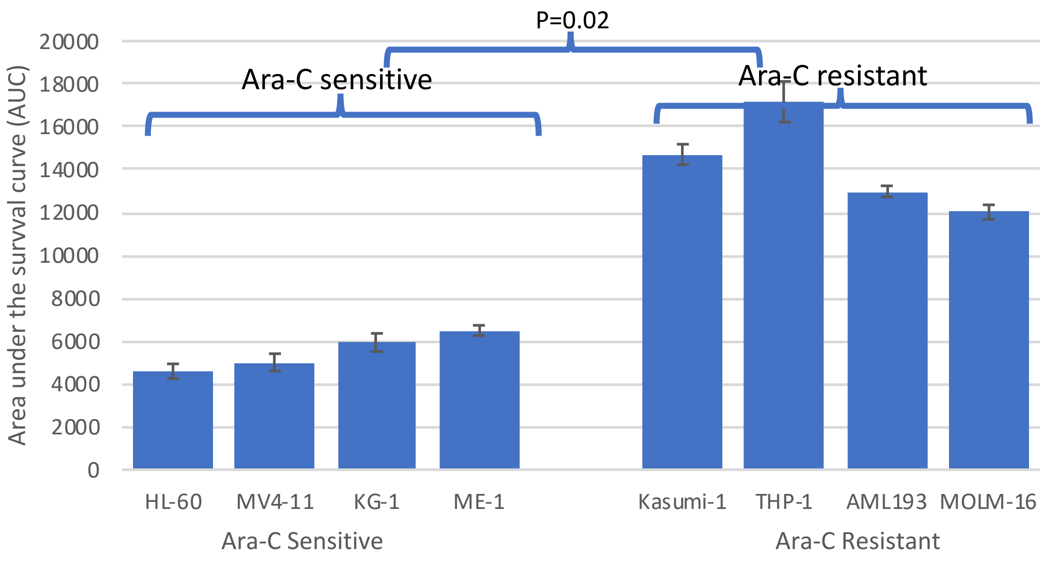

B) has-miR-24-3p levels in ara-C sensitive and resistant cell lines

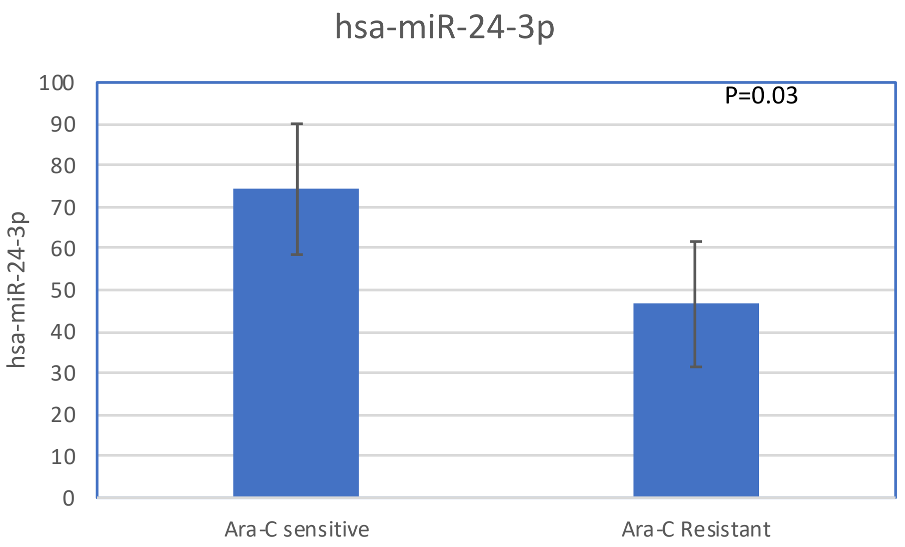

Supplementary Figure S2. A) Eight AML cell lines were treated with varying concentration of ara-C for 48 hrs followed by measuring cell viability using MTT assays using as described previously (Bhise et al, 2015). Area under the survival was calculated using Graphpad Prism and cell lines were classified as sensitive or resistant to ara-C. B) miR24-3p levels were observed to be higher in cell lines sensitive to ara-C as compared to ara-C resistant cell lines.
